# 4D nucleomes in single cells: what can computational modeling reveal about spatial chromatin conformation?

**DOI:** 10.1186/s13059-016-0923-2

**Published:** 2016-04-07

**Authors:** Monika Sekelja, Jonas Paulsen, Philippe Collas

**Affiliations:** Department of Molecular Medicine, Faculty of Medicine, University of Oslo, PO Box 1112, Blindern, 0317 Oslo Norway

## Abstract

Genome-wide sequencing technologies enable investigations of the structural properties of the genome in various spatial dimensions. Here, we review computational techniques developed to model the three-dimensional genome in single cells versus ensembles of cells and assess their underlying assumptions. We further address approaches to study the spatio-temporal aspects of genome organization from single-cell data.

## Background

Increasing evidence indicates that the spatial, three-dimensional (3D) organization of chromatin influences gene expression and cell fate [1–8]. Chromosome conformation capture (3C) techniques coupled with high-throughput sequencing enable interrogations of short-range and long-range chromosomal interactions on a genome-wide scale [8–11]. One such technique, Hi-C [8], involves crosslinking (or ‘freezing’) interacting chromosome regions, fragmentation of chromatin, ligation of the crosslinked fragments, paired-end sequencing of the ligation products, and mapping of the sequence reads to a reference genome. A matrix is constructed to map read pairs that reflect contact between two chromosome regions by binning the genome and ascribing each read pair into the corresponding bin. The frequency of read pairs in each bin reflects contact frequencies between loci. These are optionally transformed into pairwise distances and used to estimate the position of these loci in a 3D space. In order to reconstitute 3D models of chromatin, interaction frequencies can directly or indirectly be used as constraints so that genomic regions with high contact frequencies are drawn to each other in the nuclear space. To improve the accuracy of 3D models of chromatin, other constraints can potentially be incorporated into structural models based on association of chromatin with known anchors in the nucleus, such as the nuclear envelope [4, 12], nuclear pore complexes [13, 14], or nucleoli [15, 16].

Most 3D genome reconstructions are performed on cell population-averaged Hi-C contact matrices [6, 8, 17–23]. The results consistently provide a hierarchical view of folding of the genome, with chromatin divided into supra-megabase compartments of transcriptionally active or inactive chromatin (the so-called A and B compartments) [6, 8] and, within these compartments, megabase-scale topologically associated domains (TADs) [7, 24, 25]. TADs show distinct boundaries, within which loci interact more frequently with one another than with loci of adjacent TADs. Unlike compartments, which can differ between cell types, TADs are more conserved [6, 8], although chromosome topology within TADs can vary [26].

The 3D conformation of chromatin is also variable between cells in a population [27, 28], presumably as a result of asynchronous gene expression patterns, epigenetic variation, and stochastic chromatin movements [29–33]. Further complicating the issue of structural variability of genomes between cells is increasing evidence suggesting that even two copies of the same chromosome in diploid cells vary in structure [26, 34]. This problem is obviously amplified for polyploid cells, such as some cancer cell types, or if one were to investigate genome structure in polyploid organisms. As discussed in this review, computational methods have been developed to address the structural variability of genomes between subpopulations of cells. Cell-to-cell heterogeneity has also been directly captured in a pioneering study by applying Hi-C to multiple single cells [35]. Other emerging single-cell, high-throughput, sequencing-based technologies provide additional evidence for cell-to-cell heterogeneity in associations of chromatin with the nuclear envelope [36], chromatin accessibility [37–39], epigenetic states [40–44], and gene expression patterns [45, 46] (Table 1).

**Table 1:** Overview of genome-wide high-throughput sequencing-based single-cell technologies

Technology (single cell)	Information	Throughput (no. cells)	Strength	Limitation	Ref.	Year
RNA sequencing	Transcriptome	High (>1000)	Resolution	Low mRNA detection limit	[45, 46]	2009
				Amplification bias		
In situ RNA sequencing	Transcriptome with RNA localization	High (>1000)	Colocalization of locus and transcript	Time-consuming	[91, 92]	2014
				Abundance of rRNA transcripts		
				Selective towards active gene		
ChIP sequencing	Protein association with the genome	High (>1000)	Reduced cost of ChIP assay	Antibody-dependent	[40]	2015
				Data sparsity/low coverage		
Hi-C	Global chromatin contact maps	Low (<100)	Global view of the genome	Data sparsity/low coverage	[35]	2013
DamID	Lamina-associated domains	Medium (<200)	No need for antibody	Proximity assay	[36]	2015
				Relative low resolution		
ATAC sequencing	Genome accessibility	Medium (<500)	Resolution	Recovery of ATAC-seq DNA fragments	[38, 39]	2015
Bisulfite sequencing (BiS)	DNA methylation	Medium (<400)	Base resolution	DNA amplification before bisulfite conversion	[41, 43]	2014
				Cost		
Reduced-representation BiS	DNA methylation	Low (<100)	Sensitivity	Low coverage	[42, 44]	2013

Abbreviations: *ATAC* assay for transposase-accessible chromatin, *ChIP* chromatin immunoprecipitation, *rRNA* ribosomal RNA

The main purpose of single-cell genome conformation studies is to assess the heterogeneity in 3D chromatin structures between cells and, therefore, characterize the subpopulations of structures. In this review, we first address computational approaches that interrogate 3D chromatin structure from population-based studies; we evaluate their underlying assumptions and focus on how these methods tackle the cell-to-cell variability in 3D chromatin structures. We further examine challenges associated with inference of chromosome structures from single-cell interrogations. We address computational techniques enabling modeling the 3D genome over time and highlight how single-cell data might benefit this exercise. Finally, we summarize implications from applications of computational modeling to study the spatio-temporal (so-called ‘4D’) and functional aspects of genome organization.

## Assessing genome conformation in cell populations

Virtually all 3D chromosome-conformation studies are based on the analysis of millions of cells, with no obvious way to discern conformations between cells in the population. As discussed in this section, however, computational methods are very helpful in resolving this issue. Although single-cell chromosome conformation can capture cell-to-cell chromosome structural heterogeneity [35], this approach comes with its own challenges. Before discussing these challenges, we describe two main methods to infer chromatin 3D structure from Hi-C data, namely consensus methods and deconvolution methods. We further evaluate issues in addressing the heterogeneity of chromosome structures from Hi-C data averaged from ensembles of cells.

### Consensus chromosome structures inferred from population-average data

Constraint-based 3D chromosome modeling strategies primarily rely on consensus methods that aim to find a unique 3D structure averaged over many representative structures [19–23, 47] (see also recent reviews [11, 48, 49]). Consensus methods typically use a matrix of pairwise distances between genomic elements obtained by transforming Hi-C contact frequencies to visualize these elements as points in a 3D space. Many of these methods exploit the property that the distance between any two points must be smaller than or equal to the sum of distances of these two points to a third point (triangle inequality principle). However, in data from a cell population with any degree of heterogeneity, this property is not met as any given distance between two points is based on an average of multiple structures in that population [50, 51]. Thus, a structure inferred from the average of millions of cells will differ from structures derived from subpopulations of cells and will typically not represent any of the structures in individual cells [51, 52].

As a result, consensus 3D structure reconstruction methods have been devised to assess chromosomal structural heterogeneity, without estimating the population of structures per se. Semi-definite programming has been applied to identify the best consensus structure fitting Hi-C data [20]. The approach includes a test of population homogeneity that examines whether the triangle inequality assumption is met in the input distance matrix and how well the distance matrix is represented by three dimensions as opposed to a higher number of dimensions [20]. A second approach assumes that structures within TADs vary across cell subpopulations and estimates this within-TAD variation using a mixture component model [19]. In this model, each component represents a unique spatial structure of these sub-TADs, with the weight of each component defining the proportion of a given structure in the population.

The ability to evaluate heterogeneity of chromosome structures is a significant advance in genome modeling from consensus methods. However, consensus methods produce a unique structure and do not escape the (incorrect) underlying assumption that structures are homogeneous. Moreover, uncertainty remains on the source of the heterogeneity estimated: for example, a population from one cell type might be truly more heterogeneous than another or data for that cell type might simply be noisier. Thus, consensus modeling techniques do not fully capture the 3D structural heterogeneity within a cell population. They might nevertheless constitute a promising approach to unveiling 3D structures in single cells.

### Deconvolution methods identify hidden substructures

**Fig. 1:**
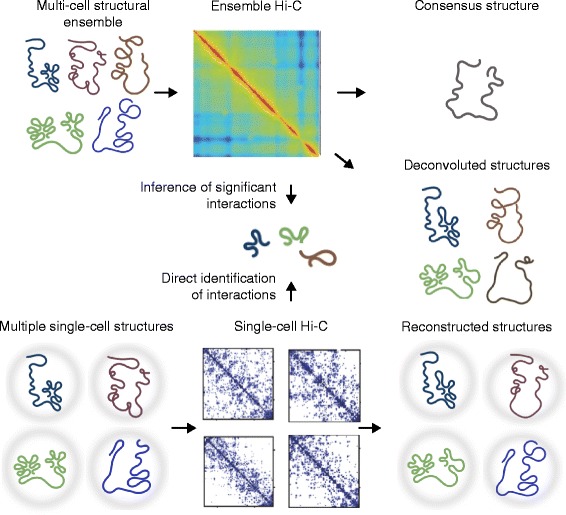
Ensemble and single-cell Hi-C computational methods. *Top*: a population of cells gives rise to a dense Hi-C heatmap, consisting of contact frequencies between all pairs of loci in the genome. The heatmap is typically colored according to the contact frequency, such that red colors indicate a high number of contacts and blue colors indicate a low number of contacts. This heatmap can be used to construct a single consensus structure or to computationally construct a set of deconvoluted structures that, in aggregate, describe the ensemble Hi-C heatmap. *Middle*: both ensemble Hi-C and single-cell Hi-C can be used to identify three-dimensional (3D) interactions between pairs of elements. For ensemble Hi-C, this is performed by using statistical models to infer significant interactions. In single-cell Hi-C, interactions are inferred directly. *Bottom*: multiple single cells are analyzed by single-cell Hi-C, giving rise to one contact matrix per cell. The contact matrix is typically visualized such that a contact is highlighted by a *blue dot*; the matrix shows sparse interaction patterns within the chromosomes. Each single-cell Hi-C contact matrix can then be used to reconstruct the corresponding 3D structures. The ensemble Hi-C heatmap is from [93]. (Single-cell Hi-C contact maps are adapted from [35])

Deconvolution methods assume that Hi-C and other 3C-based data arise from many chromatin substructures present in a cell population and seek to identify these substructures [53–57] (Fig. 1). These methods demultiplex the data to identify structurally plausible, unobserved substructures. Two different deconvolution strategies have been applied to date: structural deconvolution and matrix deconvolution.

Structural deconvolution methods occur at the 3D structure reconstruction level, applying constraints such as enforced intrachromosomal and interchromosomal interactions, nuclear boundary or volume exclusion [53, 54, 58, 59]. The constraints are applied such that the population as a whole should satisfy the constraints [54] or such that individual structures within the population should satisfy as many of the constraints as possible [58, 59]. In both approaches, the resulting structural ensemble can be clustered to study the underlying structural variability and sub-population constituents. By contrast, matrix deconvolution can be applied directly on contact frequency matrices using information from, for example, TADs [57, 60] or similar topological domains [61], to search for a set of contact frequency matrices that optimally reflects the proportions of each predicted substructure in the cell population. This set of matrices should, in aggregate, reflect the input data [57]. Illustrating this point, deconvolution of individual single-cell Hi-C contact matrices [35] pooled into one mixed matrix has been shown to successfully recover the single-cell Hi-C matrices [57]. Although matrix deconvolution is usually faster than structural deconvolution, the substructures recovered might not be physically plausible. A major drawback of all deconvolution methods is the requirement of extensive computational resources. It is also currently not clear how accurate current deconvolution methods really are and whether the current data allow for well-determined estimation of structural sub-populations. We refer to recent reviews addressing these methods in detail [11, 49, 61].

Although computationally more demanding, deconvolution techniques seeking an ensemble of 3D structural solutions are better suited than consensus methods to capture the inherent heterogeneity of chromosome structures in a cell population. This in turn raises the question of whether one can evaluate through deconvolution the extent of 3D structural heterogeneity in a given experimental system and use this information design for more-rationalized interrogations of 3D chromosome conformations: for instance, how futile is it to analyze high-throughput 3C data knowing that chromosome conformation is estimated to be heterogeneous, and should one rather consider single-cell approaches?

## Inference of 3D chromatin structure from single-cell Hi-C contact maps

A breakthrough in the field of single-cell genomics came with the report of a protocol enabling the extraction of Hi-C contact maps from single cells by allowing several steps in the protocol to occur inside intact nuclei [35]. From each extracted single-cell contact map, 3D structures can be inferred using a simulated annealing approach [35] or other reconstruction approaches [23, 34]. Interestingly, large structural differences are noted between individual cells, the greatest variance residing in inter-TAD and interchromosomal contacts, whereas TAD structures are conserved. In addition, because male cells were purposely examined in this study (male cells only bear one X chromosome), repeated 3D reconstruction of chromosome X from a given cell should result in one unique structure. This view is, however, challenged by data sparsity and noise inherent to single-cell Hi-C experiments. As discussed below, this challenge becomes greater in the reconstruction of autosomes, present in two copies in diploid cells, owing to structural differences between these two copies.

### Sparseness of single-cell Hi-C contact maps can hamper high-confidence 3D structure reconstruction

A crucial issue with single-cell Hi-C chromosome contact information is the inherent sparsity of the contact frequency maps. Approximately 2.5 % of the theoretical total possible number of ligations has been shown to be recovered in the only single-cell Hi-C study reported to date [35].

**Fig. 2:**
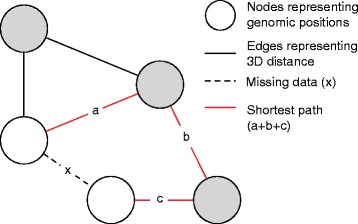
Shortest-path principle. A single-cell Hi-C matrix can be represented as a graph, where nodes (*circles*) correspond to genomic regions and edges (*black lines*) bear weights corresponding to the three-dimensional (3D) distance between the bins. For pairs of nodes with missing data (*dotted line*; *x*), a value can be estimated by finding the shortest possible path (*red edges*; *a* + *b* + *c*) between the two nodes, traversing the edges with observed distance values

One approach to alleviate the sparsity of single-cell Hi-C data is to computationally impute the “missing data”. This can be achieved by constructing a graph based on the observed contacts (edges in the graph) and computing the shortest possible path between the missing edges (where no contact is found; Fig. 2). The imputed missing distances have, in fact, the neat property that they satisfy the triangle inequality principle [23]. While this helps circumvent the missing value problem, it might, however, introduce additional noise as the imputed values are only rudimentary estimates of the true distances.

**Fig. 3:**
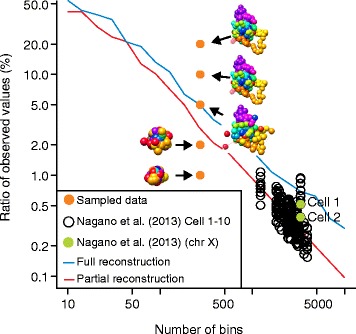
Single-cell Hi-C data sparseness. Ratio of observed values as a function of the number of bins, that is, the size of the structure being reconstructed. To assess the effect of sparseness of single-cell Hi-C data, a known structure is considered and sparse data are sampled from the structure by randomly selecting a smaller and smaller subset of the distances (20 %, 10 %, 5 %, 2 %, 1 %; orange dots). These particular structures are compared with an estimated curve showing the minimum ratio of observed values for complete reconstruction (*blue line*) or partial reconstruction (*red line*). Data from Nagano et al. [35] are shown as *black circles* and the X chromosome datasets from two individual cells (*Cell 1* and *Cell 2*) are highlighted in *green*. (Graph adapted from [34])

We recently addressed the challenge of missing values and proposed a method to down-weight contacts relative to the degree of confidence in their estimates [34]. To reconstruct 3D structures from single-cell Hi-C data, a manifold-based optimization method was used that enables incorporation of such weights. To assess the implication of data sparsity on the reconstruction of 3D structures, single-cell Hi-C contact matrices were constructed in silico where 80–98 % of the entries in the matrices were randomly deleted and the ability to reconstruct the original structure from the sparsely sampled data was examined [34]. This showed that, even with 90 % missing contacts, the reconstructed structure was essentially no different from the original structure. However, from more sparse data (>95 % missing), the similarity between the original and reconstructed structures decreases dramatically [34] (Fig. 3). Comparison of these theoretical values with existing single-cell Hi-C data shows that Hi-C data are, at the current bin size resolution (e.g., 50 kb), too sparse for high-confidence chromosome structure reconstruction, even under noise-free conditions [34]. However, by enabling increased bin sizes, the robustness of structure reconstruction can be increased, although at the cost of reduced structural resolution. Increasing coverage will, therefore, either allow for higher-resolution Hi-C maps (smaller bin sizes) or enable a greater tolerance of missing data without loss of confidence in the reconstructed 3D models.

Another important limitation to Hi-C map resolution is imposed by the genomic distribution of the digestion sites of the restriction enzyme used. To overcome this, Ma and colleagues [62] have proposed using DNase I, which cleaves the DNA into fragments smaller and more frequent than those from restriction enzymes used in conventional Hi-C protocols. The increased number of fragments leads to a higher number of possible proximity ligations and might increase Hi-C map resolution with sufficient sequencing coverage. Additionally, capture arrays can be used to increase resolution for selected loci [63, 64] at the expense of compromising full-genome reconstruction.

### Autosomal chromosomes further complicate the picture

The human genome consists of two copies of autosomal chromosomes, yet this has often been overlooked in Hi-C experiments because it is difficult to distinguish the two copies. Manifold-based optimization can successfully reconstruct 3D structures of a single X chromosome in male diploid cells from single-cell Hi-C data despite their relative noisiness and sparsity [34]. For autosomal chromosomes, however, reconstruction is less successful [34]. Simulation of a diploid situation by summing Hi-C contact maps of the X chromosome from two different individual cells reveals that 3D reconstruction is compromised. This provides important clues on specific challenges with genome 3D reconstruction in mixed Hi-C maps as even two a priori indistinguishable autosomes in single cells can hamper 3D reconstruction.

A possible solution might be to recover diploid maps based on haplotype information [6, 26, 65, 66]. This, however, requires information on allele-specific sequence variations (polymorphisms), which is typically not available for most cell lines. Haploid cell lines might also be suitable for modeling the spatial genome conformation in single cells, such as those used to map genome-wide chromatin nuclear lamina interactions [36] or multi-locus chromatin contacts [67].

## Distinguishing biological variation from technical noise

An intriguing characteristic of genome-wide 3C-based data is that the data can be used in various kinds of analyses in addition to reconstruction of 3D structures. Importantly, not all types of analyses are prone to the effects of averaging subpopulations. For instance, when one is interested in studying 3D contacts between individual loci (e.g., contacts between promoters and enhancers), the goal is to identify the statistically enriched contacts above an average background [68–71]. To do so, a model considering both the decreased chance of contacts with increasing linear genomic distance between loci and the variance of the contact frequencies is required.

A corresponding type of analysis in single-cell Hi-C would be to consider any ligation event between two restriction fragments as a contact or rely on multiple contacts in near proximity [35]. Yet, in such an analysis, how to reliably estimate the variance of contact frequencies for a given pair of loci is not clear, so it can be more appropriate to use ensemble Hi-C to study individual contacts. One way to estimate the variance in single-cell Hi-C studies could be to base the analysis on a large aggregate of multiplexed single-cell Hi-C datasets by, for example, using DNA barcoding [40]. However, this raises the question of how many single-cell datasets are required to obtain biologically relevant insights and how much information is gained from aggregated single-cell data as opposed to ensemble-cell data.

Another type of investigation is to pre-select a set of loci and consider their mutual 3D colocalization [72, 73]. In this situation, both genomic distance and variance need to be considered in order to identify statistically significant colocalizations. As multiple loci are considered, however, the effect of averaging over subpopulations again becomes a limiting factor. In single-cell Hi-C, where the effect can be avoided, the detection of multiple colocalized loci is instead hampered by the fact that only two pairs of restriction fragments can be observed for any given interaction.

## Towards the 4D nucleome

### Inferring pseudotime 3D conformational trajectories from cross-sectional data

**Fig. 4:**
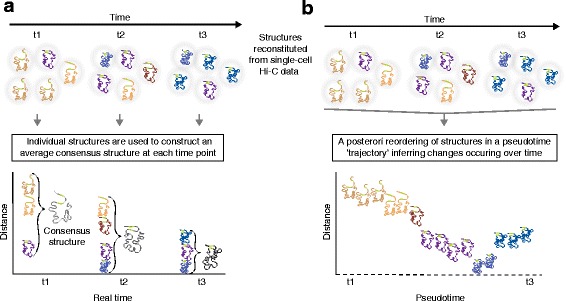
Reconstitutions of chromosome structures in a time-series analysis of three-dimensional (3D) genome conformation: consensus structures at each time-point versus reconstruction of structures through a pseudotime trajectory. **a** 3D chromosome structures determined from Hi-C data in single cells in a time-series (time-points *t1*–*t3*) can be used to determine, at each time-point, an average consensus conformation. This approach can provide information on variance, provided that sufficient numbers of single cells are analyzed. The green portions in each structure mark two loci between which distance is assessed over time. Structures occurring more than once at the same time-point are highlighted in bold. In this scenario, heterogeneity of chromosome structures in the population of single cells compromises the analysis and conceals the actual dynamics in chromatin structure. **b** A posteriori computational re-ordering of chromosome structures inferred from single-cell Hi-C data. This exercise enables the reconstruction of a pseudotime trajectory of dynamic changes of the structures between the first and last time-points at which Hi-C data are collected. Reordering of chromosome structures aids in revealing their dynamics

Increasing experimental evidence supports a view of local and global alterations in spatial genome conformation as cellular states change during development and differentiation [2, 26, 74] or in disease [75–77]. However, there are to date no truly longitudinal (developmental) studies of 3D chromatin conformation in single cells because 3C techniques are destructive to cells. Single-cell interrogations could prove useful to a posteriori recapitulate pseudo-developmental changes, or ‘trajectories’, in 3D chromosome conformation and thereby infer a pseudo-4D view of chromatin dynamics (Fig. 4). Support for this approach comes from developmental gene expression studies using single-cell RNA sequencing [78–80]. As expected from cell-to-cell heterogeneity within populations, single cells analyzed at any time-point in a time-series show variations in transcript levels [81–84]. At consecutive time-points, individual cells will be expected to show both similar and distinct transcript levels across cells. This information can be used to computationally reorder cells a posteriori in order to find a pseudotime trajectory; see especially the perspective by Trapnell [52] for an excellent assessment of this approach.

A similar strategy could conceivably be used to reorder cells in a pseudotime trajectory based on single-cell Hi-C data (Fig. 4a, b). Instead of gene expression data for each cell, information can be extracted from single-cell Hi-C contact maps to construct a matrix consisting of distances between pairs of bins in each single-cell genome. This would in principle allow an a posteriori reconstruction of a path of changes in chromatin structures between two consecutive time-points at which Hi-C data are actually collected (Fig. 4b). Such pseudotime reordering of chromatin structures from single-cell Hi-C contact maps might provide more accurate information on the specific trajectories that genome topology takes during development or differentiation.

### Locus tracking in living cells by real-time imaging

Even though high-throughput sequencing-based methods do not currently enable true 4D studies of chromatin organization in single cells, emerging microscopy-based cell-imaging techniques provide opportunities. For example, locus tracking, relying on modifications of CRISPR/Cas9-mediated genome-editing approaches to tag fluorescent molecules to specific loci, enables the visualization of movements of loci in the nuclear space in living cells [85, 86]. Targeting of a nuclease-deficient dCas9 mutant fused to a fluorophore-encoding protein [e.g. enhanced green fluorescent protein (EGFP)] to a given locus with sequence-specific guide RNAs can be achieved for intergenic repeat regions (e.g., telomeres) or genes. This approach is non-disruptive and, pending that low-intensity fluorescence emission is used to avoid photobleaching, it can be applied to track changes in locus positioning, for example, in response to a stimulus. A current limitation of this approach, however, is the relatively low fluorescence intensity of the tags, making their detection above background at single loci difficult. This often requires the use of several guide RNAs to target sufficient dCas9–EGFP molecules (or other fusions of dCas9 with fluorescent proteins) to the locus of interest [85, 86]. Continuous development of increasingly more-potent fluorophores will probably remedy this issue. Multi-color tagging of several loci simultaneously [87] in combination with super-resolution live-cell microscopy could also enable visualization of interactions between genomic regions in real time. Dynamic interactions can in turn be correlated with gene expression patterns [88]. Furthermore, new strategies for real-time simultaneous observation of gene localization and expression are emerging [89], providing some functionality to spatial locus positioning and chromosomal interactions. In addition, analysis of epigenetic states over time has been reported with the advent of live-cell imaging techniques for monitoring DNA methylation changes using a reporter-based system [90]. Although currently low throughput, these live-cell techniques do enable real-time investigations of chromatin conformation.

## Conclusions

Advancements in wet-lab high-throughput genomics and computational methods in the past 15 years have taken our understanding of the genome to a whole new level by allowing genome-wide assessments of chromatin conformation in the 3D space. Single-cell high-throughput genomics is still in its infancy and most computational techniques are developed for ensemble-cell Hi-C data. Variation is, however, an inherent property of genome structures in a cell population. We have highlighted in this review challenges in the interpretation of Hi-C data arising from this variation. As a result, a number of computational methods have been proposed to take on board this heterogeneity. Consensus methods of modeling chromatin in 3D poorly address structural heterogeneity because they produce a single chromatin structure based on data obtained from millions of cells. By contrast, deconvolution techniques, despite being computationally more demanding, enable inference of the main substructures that exist in an ensemble of cells.

To disentangle the heterogeneity in cell populations, it will be essential to seek improvements in the efficiency of laboratory methods and in the streamlining of computational techniques to explore chromatin dynamics from down-sized cell populations or from single cells. When studying chromatin dynamics in subpopulations, structures from single-cell Hi-C datasets provide more information on structural variance than deconvoluted structures from ensembles of cells, pending that hundreds of single-cell structures are determined. In time-course studies, laboratory and financial resources might rather be used to examine more time-points, albeit from down-sized cell populations. This, however, requires the development of appropriate deconvolution methods to assess the main subpopulations and their chromatin dynamics.

Arguably, the most significant weakness in single-cell Hi-C analyses to date is sparsity of the values in the distance matrix. We have shown that this data sparsity does not necessarily prevent structure modeling [65]. Yet the question remains of how to distinguish significant interactions from mere random (albeit cross-linkable) contacts in single cells. Deep sequencing of single-cell Hi-C ligation products will be necessary to enable the reconstruction of 3D chromatin conformations with high confidence.

By analogy to single-cell gene expression and pseudo-developmental trajectory reconstitutions in developmental studies, another advantage of single-cell high-throughput chromosome conformation queries is the foreseen ability to re-order single-cell structures to infer a developmental path of changes in chromatin conformation—for example, as cells go through the cell cycle or differentiate. This is expected to constitute an important step in our understanding of the spatial dynamics of the 4D nucleome. Furthermore, with the emergence of additional single-cell technologies that allow interrogations of the epigenome [40, 41, 43], chromatin accessibility [38, 39] or associations of loci with the nuclear periphery [36] (Table 1), we foresee the emergence of more-sophisticated (and arguably more accurate) models of genome architecture. Finally, fluorescent tagging of multiple loci simultaneously in single cells, even though this approach is currently not high-throughput in terms of the number of identifiable loci that can be examined, unveils possibilities to interrogate the dynamics of relative positioning of loci in real time.

We can look forward to exciting developments in the combination of high-throughput sequencing-based techniques and imaging methodologies to interrogate the functional significance of chromatin folding in space and real-time in single cells. Efficient methods to estimate heterogeneity within a given cell population and to enable integration of several types of single-cell ‘omics’ data will aid in developing improved models of genome conformation at various scales.

## Abbreviations

3C: chromosome conformation capture
3D: three-dimensional
ATAC: assay for transposase-accessible chromatin
ChIP: chromatin immunoprecipitation
DamID: Dam identification
EGFP: enhanced green fluorescent protein
TAD: topologically associated domain
